# Supplementary material for: PtrARF2.1 Is Involved in Regulation of Leaf Development and Lignin Biosynthesis in Poplar Trees
Source: Int J Mol Sci. 2019 Aug 24;20(17):4141. doi: 10.3390/ijms20174141 (PMC6747521; doi:10.3390/ijms20174141)
Supplement: Supplementary file 1 [file ijms-20-04141-s001.zip › Supplementary Figures.docx]

**Poplar PtrARF2.1 is involved in regulation of** **leaf development and lignin biosynthesis**

Yongyao Fu, Papa Win, Huijuan Zhang, Chaofeng Li, Yun Shen, Fu He and Keming Luo


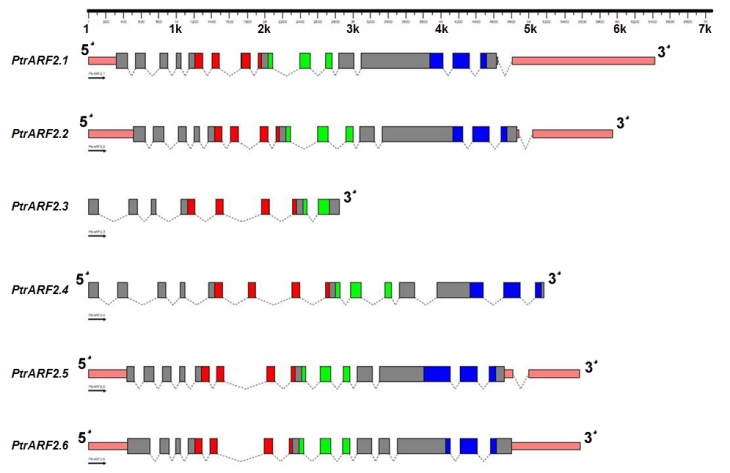


**Figure S1**. Intron-exon structure of all the *PtrARF2* genes from *P. trichocarpa* genome (V3.0)*.* Genomic structure analysis of *PtrARF2* genes were drawn using Fancy gene V1.4 software (http://bio.ieo.eu/fancygene/). The pink portion represents the 5’ and 3’ utr region; the strandlines represent intron parts; the gray boxes indicate exon parts; the red and green boxes correspond to the B3-DNA binding domain (B3-DBD) and ARF domain, respectively and the blue boxes (Aux/IAA domain) responsible for dimerization with Aux/IAA proteins.


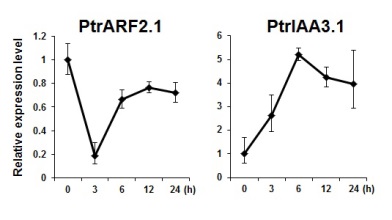


**Figure S2**. Auxin responsiveness assay of PtrARF2.1. Quantitative RT-PCR analysis of *PtrARF2.1* transcripts in total RNA sample from wild-type seedlings treated with or without 20 μm IAA for several hours. Transcript levels of *PtrIAA3.1* were used as positive controls.


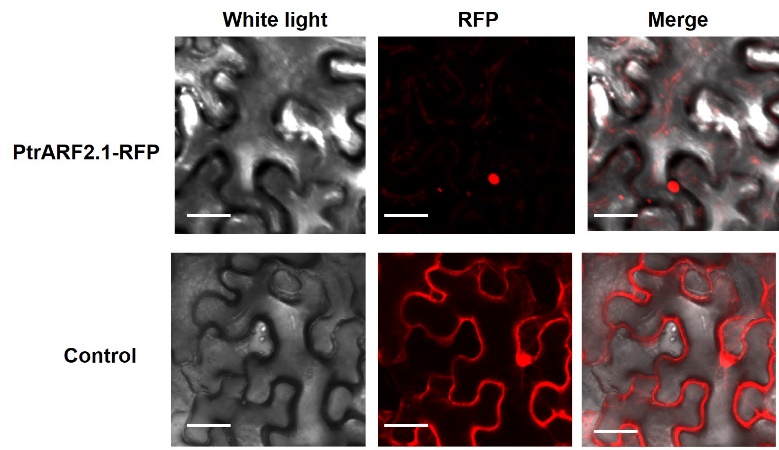


**Figure S3**. Nuclear localization of PtrARF2.1. The PtrARF2.1 protein was expressed in the nucleus of tobacco leaf epidermal cells. RFP fluorescent images were examined with a confocal microscope at 18 h after bombardment (scale bars: 50 μm).
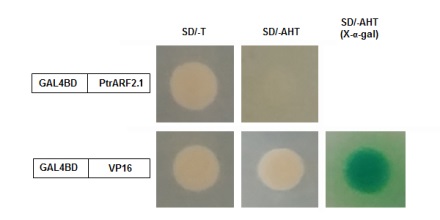


**Figure S4**. Transcriptional activity assay of PtrARF2.1. The yeast carrying GAL4BD-PtrARF2.1 cannot grow on medium without adenine (A), histidine (H) and tryptophan (T) (SD/–AHT) while the yeast carrying GAL4BD-VP16 can grow on medium SD/-AHT and induce the activity of X-α-gal as positive controls.


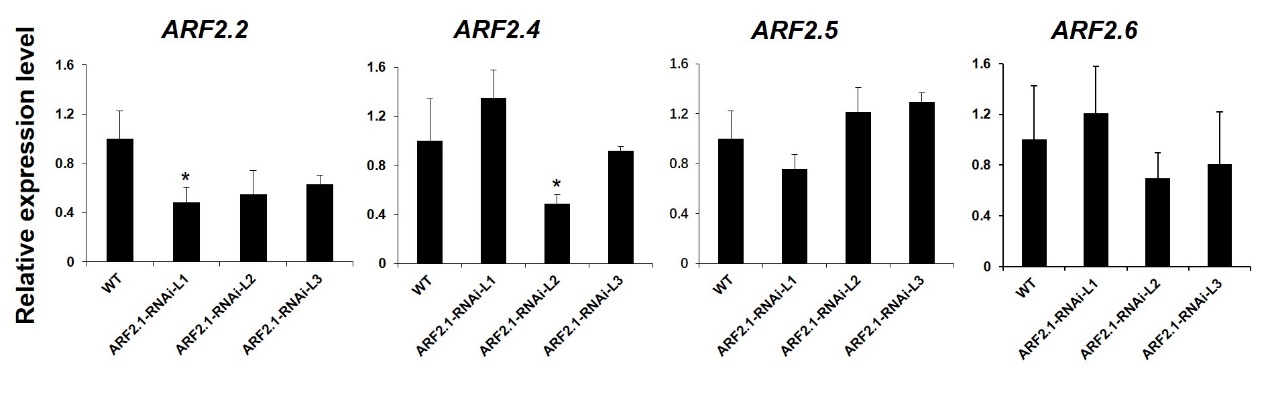


**Figure S5**. Expression analysis of other *PtrARF2* genes in *PtrARF2.1-RNAi* lines. qRT-PCR analysis of *PtrARF2.2-2.6* transcripts in three independent *PtrARF2.1-RNAi* lines while *PtrARF2.3* transcript was not detected*.* Total RNA was extracted from the leaf tissue. The poplar *18S* rRNA was used as an internal control. Error bars mean ±SD of three replicates. The asterisk indicate the statistical significance using Student’s t-test: * p-value < 0.05.

**
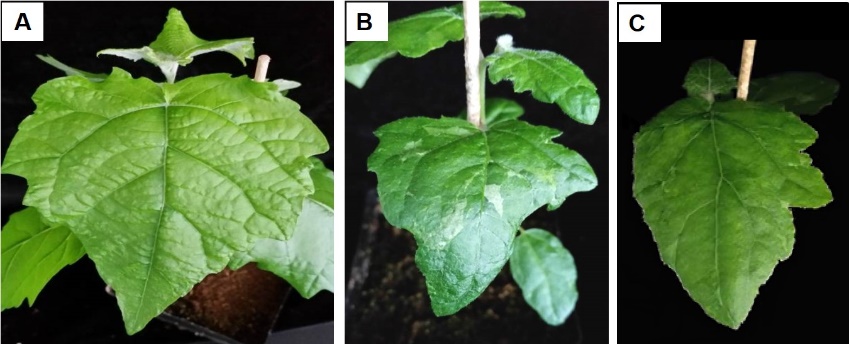
**

**Figure S6.** Altered leaf shapes in *PtrARF2.1-RNAi* transgenic poplars. Representative three-month-old leaves from the transgenic lines (B, C) in contrast to the wild-type (A).

**Supplementary Table S1**. All the primers used in this study.

**Supplementary Table S2**. Lignin content in *PtrARF2.1-RNAi* leaves.

**Supplementary Table S3**. DEGs between WT and *PtrARF2.1-RNAi* Line 1.

**Supplementary Table S4**. The DEGs encoding the members of TF families.

**Supplementary Table S5**. The DEGs involving in lignin metabolic process.
