# Supplementary material for: PtrARF2.1 Is Involved in Regulation of Leaf Development and Lignin Biosynthesis in Poplar Trees
Source: Int J Mol Sci. 2019 Aug 24;20(17):4141. doi: 10.3390/ijms20174141 (PMC6747521; doi:10.3390/ijms20174141)
Supplement: Supplementary file 1 [file ijms-20-04141-s001.zip › Supplementary Table S1.docx]

**Supplementary Table S1. Lignin content in *PtrARF2.1-RNAi* leaves**

| Poplar plants | Lignin (mg/100 mg DW) |
| --- | --- |
| WT | 3.56 + 1.56 |
| *PtrARF2.1-RNAi* L1 | 6.15 + 2.19** |
| *PtrARF2.1-RNAi* L2 | 6.13 + 1.60** |

Data indicate three independent assays. DW means “Dry Weight”. Asterisks indicate significant differences using Student’s t-test (*P < 0.05, **P < 0.01).
