# Supplementary material for: PtrARF2.1 Is Involved in Regulation of Leaf Development and Lignin Biosynthesis in Poplar Trees
Source: Int J Mol Sci. 2019 Aug 24;20(17):4141. doi: 10.3390/ijms20174141 (PMC6747521; doi:10.3390/ijms20174141)
Supplement: Supplementary file 1 [file ijms-20-04141-s001.zip › Supplementary Table S3.docx]

**Supplementary Table S3**. The DEGs encoding the members of TF families

| Gene ID | Log2ratio (ARF2/WT) | FDR | Included Domain | Description |
| --- | --- | --- | --- | --- |
| B3 |  |  |  |  |
| Potri.009G108500.v3.0 | -1.20 | 3.32E-12 | B3 | B3 domain-containing protein REM19-related |
| Potri.004G146900.v3.0 | -1.06 | 1.71E-13 | B3 | B3 domain-containing protein REM19-related |
| ERF |  |  |  |  |
| Potri.018G047300.v3.0 | 8.32 | 4.25E-10 | AP2 | Ethylene-responsive transcription factor 18 |
| Potri.010G129200.v3.0 | 2.42 | 1.26E-38 | AP2 | Similar to DNA-binding protein RAV2 (RAV2); similar to AP2 domain-containing protein RAP2.8 |
| Potri.006G261200.v3.0 | 1.91 | 3.55E-08 | AP2 | Similar to PPLZ02 protein |
| Potri.004G047600.v3.0 | 1.65 | 3.32E-05 | AP2 | AP2 domain |
| Potri.001G092400.v3.0 | 1.53 | 9.52E-09 | AP2 | Similar to AP2 domain-containing transcription factor |
| Potri.014G099900.v3.0 | 1.25 | 4.02E-13 | AP2 | Similar to AP2 domain-containing transcription factor; similar to transcription factor TINY |
| Potri.003G139300.v3.0 | 1.24 | 3.91E-10 | AP2 | Ethylene-responsive transcription factor CRF5-related |
| Potri.001G154100.v3.0 | -2.14 | 1.16E-05 | AP2 | Ethylene-responsive transcription factor 15-related |
| Potri.010G006800.v3.0 | -2.03 | 1.57E-118 | AP2 | Similar to AP2 domain transcription factor RAP2.3 |
| Potri.003G081200.v3.0 | -1.19 | 3.92E-06 | AP2 | Ethylene-responsive transcription factor 15-related |
| bHLH |  |  |  |  |
| Potri.002G180300.v3.0 | 1.92 | 2.90E-28 | HLH | Transcription factor bHLH57-related |
| Potri.003G093200.v3.0 | 1.66 | 6.26E-55 | HLH | Transcription factor bHLH71-related |
| Potri.017G081300.v3.0 | 1.61 | 1.65E-21 | HLH | Similar to supported by full length cDNA gi:26453215 from (Arabidopsis thaliana) |
| Potri.010G130000.v3.0 | 1.43 | 1.68E-17 | HLH | Basic helix-loop-helix (bHLH) family protein |
| Potri.015G104200.v3.0 | 1.23 | 1.58E-07 | HLH | Basic helix-loop-helix (bHLH) family protein |
| Potri.019G099300.v3.0 | -1.74 | 6.00E-04 | HLH | Basic helix-loop-helix domain-containing protein |
| Potri.019G089000.v3.0 | -1.54 | 2.97E-23 | HLH | Sterol regulatory element-binding protein |
| GATA |  |  |  |  |
| Potri.001G188500.v3.0 | 1.79 | 3.27E-05 | GATA | GATA transcription factor 9 |
| Potri.010G223300.v3.0 | 1.27 | 4.59E-17 | GATA | Similar to GATA transcription factor 3 |
| Potri.019G033000.v3.0 | 1.03 | 2.12E-04 | GATA | GATA binding factor |
| GRAS |  |  |  |  |
| Potri.016G027100.v3.0 | 1.27 | 2.08E-06 | GRAS | DELLA protein GAI-related |
| Potri.001G409500.v3.0 | 1.15 | 5.22E-08 | GRAS | GRAS domain family |
| Potri.001G415200.v3.0 | 1.08 | 1.19E-07 | GRAS | GRAS domain family |
| Potri.T138100.v3.0 | -3.55 | 2.63E-05 | GRAS | Protein SCARECROW |
| SRF-TF |  |  |  |  |
| Potri.002G151700.v3.0 | 2.50 | 6.03E-08 | SRF-TF | MADS box protein |
| Potri.003G170000.v3.0 | -1.89 | 4.19E-37 | SRF-TF | SRF-type transcription factor (DNA-binding and dimerisation domain) |
| Potri.T062400.v3.0 | -1.12 | 1.48E-09 | DUF260 | LOB domain-containing protein 37-related |
| MYB |  |  |  |  |
| Potri.003G144200.v3.0 | 7.29 | 5.88E-05 | Myb_DNA-binding | MYB-like dna-binding protein 123 |
| Potri.008G088000.v3.0 | 3.39 | 2.46E-04 | Myb_DNA-binding | MYB-like dna-binding protein 136 |
| Potri.007G023800.v3.0 | 3.19 | 4.31E-20 | Myb_DNA-binding | Protein DNJ-23-related |
| Potri.006G150300.v3.0 | 2.55 | 7.89E-89 | Myb_DNA-binding | Transcriptional adaptor 2 ADA2-related |
| Potri.009G116600.v3.0 | 2.38 | 2.34E-05 | Myb_DNA-binding | Similar to myb family transcription factor |
| Potri.017G082500.v3.0 | 2.27 | 1.33E-33 | Myb_DNA-binding | MYB-like dna-binding protein 81 |
| Potri.010G167500.v3.0 | 1.59 | 3.36E-07 | Myb_DNA-binding | MYB-like dna-binding protein 39 |
| Potri.001G118800.v3.0 | 1.57 | 7.04E-01 | Myb_DNA-binding | MYB-like dna-binding protein 92 |
| Potri.013G067500.v3.0 | 1.37 | 7.93E-41 | Myb_DNA-binding | MYB-like dna-binding protein 53 |
| Potri.014G022500.v3.0 | 1.23 | 2.46E-32 | Myb_DNA-binding | MYB-like dna-binding protein 173 |
| Potri.005G001600.v3.0 | 1.08 | 6.03E-11 | Myb_DNA-binding | MYB-like dna-binding protein 170 |
| Potri.006G221800.v3.0 | 1.05 | 4.30E-82 | Myb_DNA-binding | MYB-like dna-binding protein 134 |
| Potri.010G114000.v3.0 | 1.02 | 1.26E-19 | Myb_DNA-binding | MYB-like dna-binding protein 165 |
| Potri.017G130300.v3.0 | -3.38 | 2.20E-06 | Myb_DNA-binding | MYB-like dna-binding protein 152 |
| Potri.006G275900.v3.0 | -2.99 | 6.42E-133 | Myb_DNA-binding | MYB-like dna-binding protein 97 |
| Potri.013G149200.v3.0 | -2.28 | 7.43E-38 | Myb_DNA-binding | MYB-like dna-binding protein 49 |
| Potri.006G191000.v3.0 | -2.19 | 4.61E-05 | Myb_DNA-binding | Similar to myb family transcription factor |
| Potri.004G021300.v3.0 | -1.41 | 6.71E-12 | Myb_DNA-binding | Myb-like dna-binding protein |
| Potri.006G221500.v3.0 | -1.35 | 1.04E-65 | Myb_DNA-binding | MYB-like dna-binding protein 183 |
| NAC |  |  |  |  |
| Potri.019G083600.v3.0 | 6.73 | 4.04E-04 | NAM | No apical meristem protein (NAM); simiar to vascular related NAC-domain protein 7 |
| Potri.001G206900.v3.0 | 2.59 | 1.45E-09 | NAM | NAC domain containing protein 128 |
| Potri.006G209200.v3.0 | 2.58 | 1.68E-11 | NAM | No apical meristem protein (NAM) |
| Potri.007G014400.v3.0 | 2.10 | 1.08E-05 | NAM | wood-associated NAC domain transcription factor 5A |
| Potri.005G116800.v3.0 | 1.79 | 1.23E-06 | NAM | No apical meristem protein (NAM); vascular related NAC-domain protein 1 |
| Potri.004G230800.v3.0 | 1.72 | 2.85E-13 | NAM | NAC domain containing protein 35 |
| Potri.002G178700.v3.0 | 1.67 | 1.12E-02 | NAM | Wood-associated NAC domain transcription factor 2B |
| Potri.011G153300.v3.0 | 1.58 | 1.54E-08 | NAM | Wood-associated NAC domain transcription factor 1A |
| Potri.002G037100.v3.0 | 1.53 | 2.30E-04 | NAM | NAC domain containing protein 74 |
| Potri.003G113000.v3.0 | 1.39 | 2.91E-04 | NAM | NAC domain containing protein 26-related |
| Potri.003G046700.v3.0 | 1.23 | 1.25E-10 | NAM | NAC domain containing protein 35 |
| Potri.001G325100.v3.0 | 1.06 | 6.11E-05 | NAM | No apical meristem protein (NAM) |
| Potri.019G031600.v3.0 | 1.03 | 3.63E-04 | NAM | NAC domain containing protein 87 |
| Potri.011G123500.v3.0 | -2.57 | 3.96E-12 | NAM | NAC domain containing protein 3 |
| Ovate |  |  |  |  |
| Potri.005G211300.v3.0 | 1.34 | 2.53E-05 | Ovate | Ovate family protein 11-related |
| Potri.006G205500.v3.0 | 1.22 | 1.64E-16 | Ovate | Similar to hypothetical protein |
| Trihelix |  |  |  |  |
| Potri.003G195300.v3.0 | 1.41 | 7.86E-09 | Trihelix | Transcription factor GT-2 and related proteins, contains trihelix DNA-binding/SANT domain |
| Potri.001G019200.v3.0 | 1.40 | 2.91E-04 | Trihelix | F16F4.11 PROTEIN |
| WRKY |  |  |  |  |
| Potri.003G138600.v3.0 | 2.44 | 2.75E-12 | WRKY | Similar to probable WRKY transcription factor 53 |
| Potri.008G094000.v3.0 | -2.50 | 6.69E-05 | WRKY | WRKY transcription factor 57-related |
| Potri.006G263600.v3.0 | -1.44 | 3.69E-24 | WRKY | Similar to putative WRKY transcription factor |
| Potri.014G119800.v3.0 | -1.04 | 5.65E-06 | WRKY | WRKY DNA-binding domain (WRKY) |
| ZF |  |  |  |  |
| Potri.019G021400.v3.0 | 2.55 | 1.58E-04 | ZF-HD_dimer | Mini zinc finger protein 1-related |
| Potri.002G143800.v3.0 | 2.10 | 1.10E-04 | ZF-C2H2 | Zinc finger (C2H2 type) family protein; similar to C2H2 type |
| Potri.003G069700.v3.0 | 1.79 | 2.44E-04 | ZF-CCCH | Zinc finger C-x8-C-x5-C-x3-H type (and similar) (zf-CCCH) |
| Potri.003G040100.v3.0 | 1.05 | 1.11E-25 | ZF-C2H2 | Serine/Threonine-protein kinase RIO |
