# Supplementary material for: PtrARF2.1 Is Involved in Regulation of Leaf Development and Lignin Biosynthesis in Poplar Trees
Source: Int J Mol Sci. 2019 Aug 24;20(17):4141. doi: 10.3390/ijms20174141 (PMC6747521; doi:10.3390/ijms20174141)
Supplement: Supplementary file 1 [file ijms-20-04141-s001.zip › Supplementary Table S4.docx]

**Supplementary Table S4**. The DEGs involved in lignin metabolic process

| Gene ID | log2Ratio (ARF2aL1/WT) | FDR | Description | Gene Name |
| --- | --- | --- | --- | --- |
| Potri.003G188500.v3.0 | 1.81 | 9.62E-32 | 4-coumarate--CoA ligase [EC:6.2.1.12] | Ptr4CL5 |
| Potri.013G157900.v3.0 | 1.78 | 0.00E+00 | Trans-cinnamate 4-monooxygenase [EC:1.14.13.11] | PtrC4H2 |
| Potri.019G130700.v3.0 | 1.59 | 6.80E-303 | Trans-cinnamate 4-monooxygenase [EC:1.14.13.11] | PtrC4H1 |
| Potri.009G095800.v3.0 | 1.18 | 2.06E-65 | Cinnamyl-alcohol dehydrogenase [EC:1.1.1.195] | PtrCAD1 |
| Potri.007G016400.v3.0 | 1.13 | 1.22E-12 | Ferulate-5-hydroxylase [EC:1.14.-.-] | PtrF5H2 |
|  |  |  |  |  |
| Potri.001G341600.v3.0 | 4.29 | 8.20E-05 | Laccase [EC:1.10.3.2] | PtrLAC5 |
| Potri.008G073700.v3.0 | 2.67 | 4.95E-06 | Laccase [EC:1.10.3.2] | PtrLAC17 |
| Potri.010G193100.v3.0 | 2.25 | 1.89E-16 | Laccase [EC:1.10.3.2] | PtrLAC28 |
| Potri.004G156400.v3.0 | 1.78 | 2.26E-04 | Laccase [EC:1.10.3.2] | PtrLAC9 |
| Potri.009G042500.v3.0 | 1.77 | 8.62E-04 | Laccase [EC:1.10.3.2] | PtrLAC21 |
| Potri.016G112000.v3.0 | 1.74 | 4.04E-43 | Laccase [EC:1.10.3.2] | PtrLAC40 |
| Potri.011G120200.v3.0 | 1.71 | 2.75E-14 | Laccase [EC:1.10.3.2] | PtrLAC29 |
| Potri.016G112100.v3.0 | 1.53 | 5.31E-12 | Laccase [EC:1.10.3.2] | PtrLAC41 |
| Potri.011G120300.v3.0 | 1.38 | 2.38E-28 | Laccase [EC:1.10.3.2] | PtrLAC30 |
| Potri.001G054600.v3.0 | 1.17 | 2.23E-04 | Laccase [EC:1.10.3.2] | PtrLAC1 |
| Potri.007G023300.v3.0 | 1.12 | 9.91E-12 | Laccase [EC:1.10.3.2] | PtrLAC16 |
| Potri.019G083600.v3.0 | 6.73 | 4.04E-04 | Wood-associated NAC domain transcription factor 6B | PtrWND6B |
| Potri.007G014400.v3.0 | 2.10 | 1.08E-05 | Wood-associated NAC domain transcription factor 5A | PtrWND5A |
| Potri.002G178700.V3.0 | 1.67 | 1.12E-02 | Wood-associated NAC domain transcription factor 2B | PtrWND2B |
| Potri.011G153300.v3.0 | 1.58 | 1.54E-08 | Wood-associated NAC domain transcription factor 1A | PtrWND1A |
| Potri.003G113000.v3.0 | 1.39 | 2.91E-04 | Wood-associated NAC domain transcription factor 4B | PtrWND4B |
| Potri.001G118800.v3.0 | 1.57 | 7.04E-01 | Myb transcription factor family protein | PtrMYB092 |
| Potri.005G001600.v3.0 | 1.08 | 6.03E-11 | Myb transcription factor family protein | PtrMYB170 |
| Potri.017G130300.v3.0 | -3.38 | 2.20E-06 | Myb transcription factor family protein | PtrMYB152 |
